# Monitoring the burden of disease in Scotland and the contribution of risk factors

**DOI:** 10.1093/eurpub/ckac129.760

**Published:** 2022-10-25

**Authors:** E Fletcher, I Grant

**Affiliations:** Data Driven Innovation Directorate, Public Health Scotland, Edinburgh, UK

## Abstract

**Background:**

The Scottish Burden of Disease (SBoD) Study monitors the contribution of over 100 diseases and injuries to the population health in Scotland, in the context of disability-adjusted life years (DALYs). Providing robust estimates of burden is the first step in identifying areas of prevention which could have the biggest impact on health; including identification of modifiable risk factors and changes in the underlying risk factor prevalence. Our aim was to estimate DALYs for 2019, to describe the current burden in Scotland and as a baseline for future burden scenarios.

**Methods:**

The SBoD 2016 study estimated the burden using routine data and patient-level record linkage. For this update, years lived with disability were estimated using 2016 age-sex-deprivation specific rates, assuming no change in disease prevalence from 2016, but taking account of changes to the population structure. Years of life lost were calculated from 2019 observed deaths and the application of the Global Burden of Disease (GBD) aspirational life table. Population attributable fractions (PAFs) were sourced from the GBD 2019 and risk factor prevalence from the Scottish Health Survey.

**Results:**

In 2019 the leading causes of burden were ischaemic heart disease (IHD), Alzheimer's/other dementias, lung cancer, drug-use disorders and cerebrovascular disease, representing over a quarter (27%) of the total DALYs in Scotland. Application of PAFs shows that a proportion of the burden for each of these causes can be attributed to modifiable risk factors.

**Conclusions:**

IHD continues to be the leading cause of health burden in Scotland in 2019. However recent years show an increase in burden of social causes and diseases affecting the ageing population. Application of PAFs demonstrate the importance of continuing to monitor both the burden of disease in Scotland and the prevalence of risk factors, to provide robust evidence for planning of local and national services.

**Key messages:**

• The Scottish Burden of Disease continues to monitor the population health landscape of Scotland.

• Ischaemic heart disease continues to be the leading cause of burden in Scotland.

